# Omega-6: Its Pharmacology, Effect on the Broiler Production, and Health

**DOI:** 10.1155/2023/3220344

**Published:** 2023-03-01

**Authors:** Mohamad Yusril Nur Mahendra, Juriah Kamaludeen, Herinda Pertiwi

**Affiliations:** ^1^Department of Health Studies, Faculty of Vocational Studies, Airlangga University, Jalan Dharmawangsa Dalam 28-30, Surabaya 60286, Indonesia; ^2^Department of Animal Science and Fishery, University Putra Malaysia, Bintulu Serawak Campus, Nyabau Road, Serawak 97008, Bintulu, Malaysia; ^3^Institute of Tropical Agriculture and Food Security, Universiti Putra Malaysia, Serdang 43400, Selangor, Malaysia

## Abstract

Lipids and oils are the primary sources of monounsaturated and polyunsaturated fatty acids (MUFA and PUFA), which are necessary for human and animal health. Omega-3 and omega-6 are essential nutrients for broilers. Omega-6 members, such as linolenic acid, are essential for broilers and must be obtained through feed. Vegetable oils are the primary source of omega-6 added to broiler feeds. Unsaturated fatty acids are better digested and absorbed than saturated fatty acids and generate more energy at a lower cost, boosting productivity. Feeding supplements with omega-6 can increase the fatty acid content in meat and increase weight, carcass, viscera, and FCR. The quality of meat taste and antioxidant content was also improved after giving omega-6 and influencing mineral metabolism. Broiler reproductive performance is also enhanced by reducing late embryonic mortality, hence enhancing fertility, hatchability, sperm quality, and sperm quantity. Meanwhile, for broiler health, omega-6 can lower cholesterol levels, triglycerides, very low-density lipoprotein, and low-density lipoprotein. It also supports support for T-helper cell (TH)-2-like IgG titers, increasing prostaglandins, eicosanoids, and antioxidants. In addition, it also supports anti-inflammation. Other researchers have extensively researched and reviewed studies on the effects of omega-6 on poultry. Meanwhile, in this review, we provide new findings to complement previous studies. However, further studies regarding the effects of omega-6 on other poultry are needed to determine the performance of omega-6 more broadly.

## 1. Introduction

The proportion of poultry meat in the average global output of 323.25 million tons (mt) over the past five years was 122.82 million tons (mt) or 37.99% [1]. Also, chicken meat output has increased in developed and developing nations over the past six decades [2]. Moreover, due to its high protein, low-fat content, and tasty flavour, chicken is expected to be the most consumed animal protein in the world in 2020. Fat and oil are frequently added to poultry diets to boost their energy density. By selecting minerals and supplements for live birds, it is possible to boost the nutritional value of chicken meat, which is one of its benefits. In recent years, numerous oils have been employed commercially to supply lipids to chickens. Some studies have indicated that supplementing poultry diets with lipids alters feed intake, energy efficiency, the profile of thigh and breast muscles, and broiler meat quality [3–5].

The supplementation of polyunsaturated fatty acids (PUFAs) can raise the concentration of PUFAs in the carcass. Fatty acids, particularly essential fatty acids, are gaining relevance in poultry feeding systems because they improve birds' health and productivity. Our health-conscious culture favours well-balanced diets to reduce the risk of unfavourable health effects [6]. PUFA has also boosted the demand for animal diets containing *γ*-linolenic acid [7]. *γ*-linolenic acid (C18 : 3 *n* − 6) improves chicken health by acting as an anti-inflammatory, antithrombotic, antiproliferative, and lipid-lowering agent by conversion to prostaglandin E1 [8].

Enriching broiler chicken muscles with PUFAs, particularly omega-3 and omega-6 fatty acids, can reduce the risk of cardiovascular disease and protect against atherosclerosis and coronary heart disease by lowering cholesterol and low-density lipoprotein (LDL) levels in the blood and reducing platelet aggregation [9]. However, there is limited research on the particular mechanism of omega-6 in broiler performance. The current article includes an update on the therapeutic qualities of omega-6, as well as its origins, chemistry, biosynthesis, absorption, distribution, broiler production, and health.

## 2. Data Collection

Data gathering a search of electronic databases follow a previous report such as PubMed, Elsevier, ResearchGate, and Google Scholar using the keywords “omega-6,” “omega-6 pharmacology,” “omega-6 absorptions,” “omega-6 for poultry,” “omega-6 for broilers,” “omega-6 for broiler production performance,” and “omega-6 for broiler health.” Selected papers from 2006 to 2022 were chosen based on their content. Relevant articles that used the keywords mentioned previously and written in English have been included.

### 2.1. Sources and Chemistry of Omega-6

Lipids' physical and chemical properties are dictated by their fatty acid content, carbon chain length, and degree of saturation. Unsaturated denotes the presence of one or more double bonds, whereas saturated indicates the lack of double bonds in chemical structure [10]. Increasing the length of the carbonic chain of saturated fatty acids raises the fat's melting point, while the presence of a double bond lowers the fat's melting point [11]. Additionally, the shape of the double bond impacts the melting point. The melting point of trans fatty acids is higher than that of their cis isomers [12].

The acyl chain of polyunsaturated fatty acids has two or more methylene-interrupted double-bond desaturations [13]. PUFAs may also contain a carboxylic acid at one end of the molecule and a methyl group at the other. This structure is named Omega (“*Ꞷ*” or “*n*”) and is subdivided into *n* − 3, *n* − 6, *n* − 7, and *n* − 9 fatty acids, which correspond to the double bond if unsaturation is present [14]. (*n*−) indicates the position of the carbon double bond counting from the methyl end. Omega-3 and Omega-6 family members are the nutritionally essential PUFAs for poultry health [15]. As seen in Table 1, there are numerous Omega-6 variants. Palmitoleic acid and oleic acid could be generated in the body via metabolic pathways. However, linolenic acid and linoleic acid are necessary fatty acids that must be ingested [14]. Additionally, high amounts of polyunsaturated fatty acids undergo autoxidation far more rapidly than saturated PUFAs, particularly when exposed to heat, light, oxygen, and transition metals during manufacture, processing, and storage [15, 16]. However, conjugated linoleic acids are sometimes misclassified as omega-6 (abbreviated −6 or *n* − 6) fatty acids. Conjugated linoleic acids are a class of fatty acids including up to 56 isomers with conjugated (juxtaposed or adjacent) double bond pairs along octadecadienoic (18 : 2) [17, 18].

**Figure 1:**
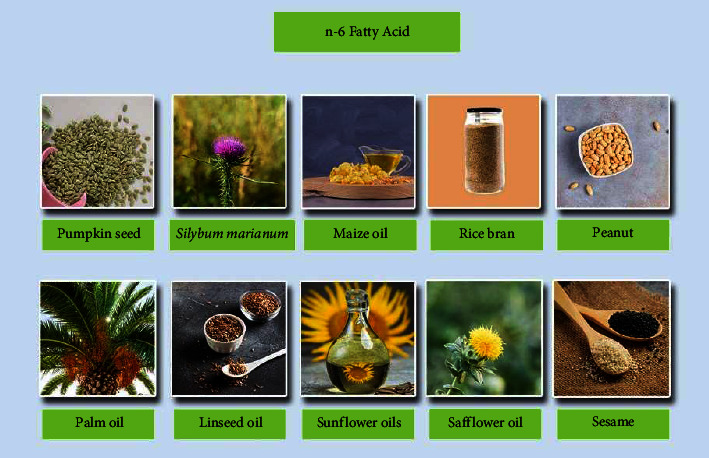
Various plants that contain a high level of omega-6.

Typical vegetable oils such as sunflower oil, safflower oil, palm oil, *Silybum marianum* oil, sesame oil, pumpkin seed oil, peanut oil, wheat germ oil, rice bran oil, linseed oil, and maize oil are sources of *n* − 6 PUFAs [19–23].  Figure 1 shows sources of *n* − 6 PUFAs. The majority of PUFAs in plants and marine foods are cis-configured. *n* − 6 PUFAs are predominantly composed of linoleic acid (C18 : 2) and arachidonic acid (AA, C20 : 4) [24], whereas linoleic acid might undergo desaturation and elongation to produce arachidonic acid (ARA, 20: 4*n* − 6) and docosahexaenoic acid (DTA, 22 : 4*n* − 6) [25].

In addition, Certık et al. [26] identified oleaginous lower filamentous fungi as a rich source of *γ*-linolenic acid. Utilizing these fungi in a solid-state fermentation method generates a bioproduct enriched with *γ*-linolenic acid that can be utilized directly as a chicken feed supplement. However, there are limited *γ*-linolenic acid sources, notably in the plant (e.g., blackcurrant, evening primrose, borage, or hemp seeds). Utilizing solid-state fermentation (SSF) is an alternate method for producing *γ*-linolenic acid from microorganisms. SSF is a prospective bioprocess that combines fungal consumption (*Thamnidium elegans*, *Cunninghamella species*, or *Mortierella isabellina*) of moist solid materials (agricultural byproducts) with the generation of valuable metabolites in a cost-effective manner [27].

### 2.2. Omega-6 Biosynthesis, Absorption, and Distribution

**Figure 2:**
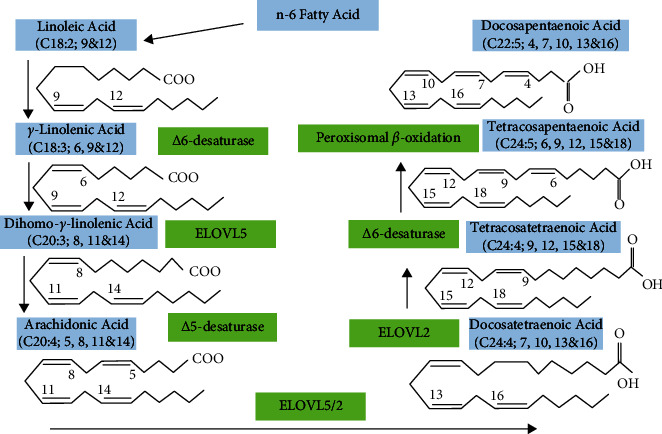
Biosynthesis omega-6.

Specifically, long-chain *n* − 6 and *n* − 3 PUFAs are regarded as necessary due to the inability of avian species to insert a double bond beyond 19 carbons due to a lack of 1–12 and 15 desaturases; they must be supplied from the food [28, 29]. Long-chain PUFAs are mainly generated in the liver [20]. During the conversion of *γ*-linolenic acid to eicosapentaenoic acid or docosahexaenoic acid and linoleic acid to arachidonic acid, desaturation and elongation of the respective precursors take place in the presence of elongation of very long-chain fatty acids ELOVL2 and ELOVL5, Δ5-desaturase, Δ6-desaturase, and peroxisomal *β*-oxidation to acquire docosahexaenoic acid (Figure 2.) [12]. However, desaturase enzymes for omega-3 and omega-6 routes are identical [29].

Absorbed *γ*-linolenic acid fatty acids and linoleic acid are transferred to adipose tissue and other tissues. In contrast, arachidonic acid is retained more in the liver, duodenum, heart, spleen, brain, and other cells (thrombocytes, peripheral blood mononuclear (PBMN)) [30]. Moreover, long-chain unsaturated fatty acids have more potential to form micelles. They could function synergistically in the absorption of saturated fatty acids (SFA) when combined with saturated fatty acids (SFA). Furthermore, micelles have an estimated particle size between 30 and 40 Å, which is sufficiently tiny to pass between the microvilli of mucosal cells [31]. In monogastric animals, fat absorption occurs between the end of the duodenum and the end of the ileum [32].

On the contrary, when *γ*-linolenic acid-rich oils are consumed orally, *γ*-linolenic acid is readily absorbed and initially appears in serum phospholipids. The substance is then dispersed across different phospholipid fractions following continued dosing. A portion of the *γ*-linolenic acid received is oxidized. The remainder is rapidly lengthened to Dihomo-*γ*-linolenic acid in the plasma, renal artery, liver, and aorta and could also elevate arachidonic acid, although exclusively in the plasma and liver [33]. Dihomo-*γ*-linolenic acid and *γ*-linolenic acid levels in the liver were proportional to the amount of *γ*-linolenic acid present, regardless of the oil source, indicating that oils are efficiently absorbed and that the amount of *γ*-linolenic acid absorbed is dose-dependent [34].

### 2.3. Effect of Omega-6 in the Broiler Production

Providing a lipid diet with the required fatty acid profile for the resultant tissue makes it possible to modify the fatty acid profiles of broiler tissues. Velasco et al. [35] showed greater feed efficiency in chicks that received diets rich in unsaturated fat sources than in chicks that were fed diets rich in saturated fat. Moreover, current poultry feed is based on grains with a high ratio of *n* − 6 fatty acids to *n* − 3 fatty acids. This feed results in high levels of arachidonic acid (20 : 4*n* − 6) in meat and egg products and reduced levels of docosapentaenoic (DPA, 22 : 5*n* − 3), eicosapentaenoic (EPA, 20 : 5*n* 3), and docosahexaenoic (DHA, 22 : 6*n* − 3) acids [23]. Furthermore, broilers fed diets with high levels of linoleic acid consumed less feed per day than those who received neither a supplement nor diets with low levels of linoleic acid [36].

Omega-6 supplementation shows positive results on broiler performance. The highest body weight, carcass yield, and FCR were observed when linoleic acid was added to broiler feed [37]. Pirzado et al. [38] also found the same result and observed that broilers' feed conversion ratio (FCR) values were significantly enhanced after receiving omega-6. With the addition of linoleic acid, a more significant concentration of chlorides was also discovered in the chickens' serum, which may be connected with a higher requirement for the concentration of HCl in the stomach in response to a higher lipid intake and enhanced chloride ion management in the body [39].

Broiler offal is also affected by omega-6 supplementation. According to a study by Gaad et al. [36], omega-6 increases the weight of giblets; the liver, heart, and gizzard are much heavier. Moreover, the comparatively high concentrations of *n* − 6 PUFAs (up to 45.0% in a corn oil diet) made cardiac and hepatic tissues the wealthiest types of fatty acids [40]. In other poultry species, dietary 6% PUFA originated from corn oil in Japanese quail showed increased productivity, follicular hierarchy in the ovarium, and the heart weight without harming other visceral organs due to its beneficial effects as an energy and essential fatty acid source, antioxidant, antiparasite, and endocrine hormone precursor [41–43]. Furthermore, the antioxidant capacity of broiler breast meat was enhanced by a diet including *γ*-linolenic acid and linoleic acid, as demonstrated in a prior study [44]. However, Fejerčáková et al. [33] discovered that GPx activity evaluated in the liver is essentially unaffected by agrimony and -linolenic acid-containing diets.

Omega-6 can also impact the fat content of poultry. According to research (El-Katcha), excessive treatment with *n* − 6 fatty acids boosts fatty acid oxidation and hence increases the metabolic rate of animals. Qi et al. [45] observed that dietary *n* − 6/*n* − 3 PUFA (10 : 1) had a substantial effect on subcutaneous and intramuscular fat content as well as meat quality in chickens (colour and tenderness). Analysis of the chemical composition revealed that hens fed a meal supplemented with linoleic acid had a higher fat content in the breast and thigh [39]. The addition of linoleic acid to compound feed for broilers, according to another study by Haščík et al. [46], enhances the intensity of growth and the proportion of internal, subcutaneous, and intramuscular fat.

Moreover, the chosen cereal product with a greater concentration of *γ*-linolenic acid (3.676 1.09 kg^−1^ in wheat bran) increased the concentration of *γ*-linolenic acid in the lipids of chicken breasts produced [47]. On the contrary, Oliveira et al. [48] emphasize the significance of *γ*-linolenic acid as a representative of *n* − 6 PUFAs, which has a synergistic effect with *n* − 3 PUFAs such as DHA and EPA, whereas dihomo-*γ*-linolenic acid and arachidonic acid possibly had a higher concentration due to a higher fraction of *γ*-linolenic acid. However, Khatibjoo et al. [49] report that a high concentration of linoleic acid in the meat of broilers fed linoleic acid could reduce the proportion of monounsaturated fatty acids and increase the proportion of polyunsaturated fatty acids.

Another study by El-Zenary et al. [50] revealed that the overall *n* − 6 PUFA content of boneless, skinless breasts mirrored that of linoleic acid in the diet. Total *n* − 6 PUFAs were highest in birds, primarily due to a higher conversion of linoleic acid to arachidonic acid. The *n* − 6 PUFAs or their sources, such as fish oil, palm oil, soybean oil, and linseed oil, also promote bone formation, development, and growth by enhancing mineral metabolism, particularly that of calcium, zinc, and magnesium, which renders them inaccessible after age [20].

The data revealed that the arachidonic acid content of *Hinai jidori* flesh can be increased with arachidonic acid dietary supplements and that *Hinai jidori* meat and soup with a higher arachidonic acid content had a significantly better taste perception than those with a low arachidonic acid content [6]. Arachidonic acid stimulates the TRPM5 cation channel, a component of type II receptor cells' sweet, umami, and bitter taste pathways, as suggested by Liu et al. [51]. Takahashi et al. [6] have demonstrated that the concentration of arachidonic acid in chicken flesh may be altered through dietary supplementation with arachidonic acid (AA) and genetic selection utilizing the polymorphism of the FADS1 and FADS2 genes as selection markers. These techniques enhance the flavour of the chicken.

In addition, it was determined that the inclusion of 2% of various sources of omega-6 fatty acids (particularly flax seed oil) in the diets of broiler breeders might reduce late embryonic mortality, hence enhancing fertility, hatchability, sperm quality, and sperm quantity [20, 52]. In addition, *n* − 6 FA-rich diets had a beneficial impact on semen volume and total spermatozoa count but a detrimental impact on spermatozoa concentration. Furthermore, avian sperm often contains a high proportion of PUFA, especially *n* − 6 PUFA [49].  Table 2 displays the effects of various plant feed resources with the highest omega-6 content on the performance of broilers. Generally, omega-6 supplementation improves broiler performance by increasing body weight and internal organs, increasing the number of fatty acids in meat, influencing mineral metabolism, and enhancing reproductive performance.

### 2.4. Effect of Omega-6 on the Broiler Health

There are limited studies on the immunomodulatory effect of PUFAs on the phagocytosis mechanism. [2]. It is known that the critical omega-6 series (particularly linoleic acid and arachidonic acid) are required for development and growth and have an essential role in the prevention and control of hypertension, arthritis, cancer, cardiovascular disease, diabetes, and autoimmune diseases [30]. In animal lipids, arachidonic acid is a polyunsaturated fatty acid (PUFA). Arachidonic acid is at the head of the “arachidonic acid cascade,” which consists of about 20 distinct eicosanoid-mediated signalling pathways that regulate a vast array of cellular processes, including those governing inflammation, immunology, and the central nervous system [71].

Plasma levels of cholesterol, low-density lipoprotein, very low-density lipoprotein, and triglycerides in broilers fed sunflower oil (containing 62.2% *n* − 6 PUFAs) were dramatically reduced. This conclusion was consistent with the findings of Shearer et al. [72] and Sidik et al. [73] who showed that the introduction of PUFAs-rich oil in broiler diets lowered serum levels of total cholesterol, very low-density lipoprotein (VLDL), and triglycerides (TG). Moreover, Bartkovský et al. [74] reported that oil containing *γ*-linolenic acid considerably decreased the serum content of triacylglycerols, cholesterol, and phospholipids compared to palm or safflower oils. In addition, the other *n* − 6 polyunsaturated fatty acids diminish the cholesterol level in plasma, with *γ*-linolenic acid being 170 times more efficient than linoleic acid. Furthermore, dietary PUFAs diminish intestinal cell chylomicron secretion and suppress hepatic fatty acid synthesis and TG production [22]. In addition, the associations between omega-6 and hypocholesterolemic index were relatively positive but moderately negative with total cholesterol and atherogenic index [3]. However, excessive omega-6 fatty acids are associated with an increased risk of severe disorders such as depression and cardiovascular disease [20].

Omega-3 and omega-6 fatty acids are essential for immunity in chicks throughout the early stages of life because of their function in cellular immunity, humoral immunity, and inflammatory regulation [75]. A high concentration of *n* − 6 PUFA may promote the helper cell (TH)-2-like response at the expense of the helper cell (TH)-1-like response [49]. Moreover, an increase in *n* − 6 PUFA inhibits the immune response to a TH-1 antigen. Also, hatching eggs from hens fed diets with sunflower oil (linoleic, *n* − 6) or linseed oil (Arachidic acid) at various ratios found that hens fed diets with a linoleic: arachidic ratio of 0.8 : 1 increased bovine serum albumin-specific IgG titer [21]. Immunoglobulin G (IgG) is the primary antibody detected in the blood of chicks and the predominant antibody generated during humoral responses [76].

Long-chain PUFAs such as eicosapentaenoic acid and arachidonic acid are precursors for eicosanoids such as prostaglandins (PGs) and thromboxanes. In line with this, Bartkovský et al. [74] reported that linoleic acid could increase tissue levels of prostaglandin E1 (PGEI) and reduce chronic inflammation. As a result, it can inhibit the release of LBT4 from polymorphonuclear neutrophils. Moreover, linoleic acid and subsequent *γ*-linolenic acid, dihomoc-linolenic acid, and arachidonic acid are essential for synthesizing the physiologically active metabolites prostaglandins [77]. Prostaglandins have a crucial role in animal autocrine and paracrine cellular interactions. Pigs are implicated in the activation and regulation of immunological responses, as indicated [78]. Furthermore, the *n* − 6 arachidonic acid metabolite prostaglandin E2 (PGE2) enhances the humoral component of the immune response and, similar to other *n* − 6 fatty acids, may reduce the cellular responses [49].

The synthesis of PGs was initiated by arachidonic acid through the conversion of COX (1-2) to PGH2, followed by the response of several PG synthases, and the transformation of PGH2 into prostanoid end products [75]. On the contrary, the immunomodulatory action of PUFAs in birds results from intercellular communications and signals that influence the responsiveness of leukocytes due to antigenic motivation [24]. This effect is strongly related to the downregulation or upregulation of many cytokines that affect the avian immune system, including IL-1, IL-2, IL-4, IL-1, IFN, and MGF-22.

Eicosanoids are lipid mediators of inflammation that are generated via the cyclooxygenase (COX) and lipoxygenase (LOX) pathways, which use arachidonic acid and eicosapentaenoic acid as substrates [79]. Meanwhile, reducing the ratio of *n* − 6 to *n* − 3 PUFAs decreases proinflammatory *n* − 6 PUFA-derived cytokines such as IL-6 [80]. Also, gamma fatty acids are recently developing biomaterials produced from lipids that can aid in preventing metabolic illness and inflammation and enhancing lipid metabolism [81, 82]. However, according to Schmitz and Ecker [83], omega-3 fatty acids inhibit the production of inflammatory genes, whereas omega-6 fatty acids have the reverse effect. Moreover, the feeding combinations, including microbially produced *γ*-linolenic acid and plant extracts, are recognized for their antioxidant and anti-inflammatory activities, such as *Agrimonia eupatoria* L. [82]. Concomitant administration of *γ*-linolenic acid could have also increased dihomo-*γ*-linolenic acid, which would compete with arachidonic acid and favour the release of less proinflammatory eicosanoids [74].

Meanwhile, linoleic acid has an anti-inflammatory impact by lowering the release of interleukin (IL)-6 and -1 and tumour necrosis factor, as demonstrated by Jung et al. [44]. Furthermore, the products of *γ*-linolenic acid metabolism influence the expression of several genes via gene product regulation. These gene products are essential for apoptosis [74].

Omega-6 could also decrease lipogenesis and increase fatty acid oxidation in the liver [84]. As a PUFA with three double bonds, *γ*-linolenic acid is vulnerable to oxidation and peroxide production. In liver cells, *γ*-linolenic acid supplementation can result in oxidative alteration and aggregation of apo B, which is then lysed [33]. Linoleic acid, another Omega-6, is a PUFA that can create multiple types of free radicals and can accelerate lipid oxidation [44]. The amount of *γ*-linolenic acid paired with agrimony extract supplementation during the 42 weeks did not affect mitochondrial SOD, GPx, GR, or GSH levels [33]. In addition, increased omega-6 consumption under stressful conditions might raise oxidative stress and a proinflammatory state, increasing the risk of atherosclerotic cardiovascular disease [85].

On the contrary, *N* − 6 fatty acids depress the immunological system [53]. In addition, the connection between *n* − 6 PUFAs and cytotoxic cell activity was found to be positive in the study [45]. Moreover, omega-6 also boosts IgG titers, prostaglandins, and eicosanoids and decreases cholesterol, triglycerides, VLDL-C, and LDL. Omega-6 supplementation had a favourable effect on the health of broilers; however, it has a pro-anti-inflammatory impact.

## 3. Conclusion

An omega-6-rich diet can improve broiler performance, including body weight, FCR, ADG, carcass, and meat quality by optimizing antioxidant activity to maintain normal metabolism. Broiler reproductive performance is also enhanced by reducing late embryonic mortality, hence enhancing fertility, hatchability, sperm quality, and sperm quantity. Meanwhile, for broiler health, omega-6 can lower cholesterol levels, triglycerides, very low-density lipoprotein, and low-density lipoprotein. It also supports support for T-helper cell (TH)-2-like IgG titers, increasing prostaglandins, eicosanoids, and antioxidants. In addition, it also supports anti-inflammation.

## Figures and Tables

**Table 1:** Various forms of the omega-6 group.

Omega family	Common name	Systematic name	*n* and Δ abbreviations
*n* − 6	Linoleic acid (LA)	*all*-*cis*-9,12-octadecadienoic acid	18 : 2*n* − 6 or 18: 2^Δ9,12^
	*γ*-Linolenic acid (GLA)	*all*-*cis*-6,9,12-octadecatrienoic acid	18 : 3*n* − 6 or 18: 3^Δ6,9,12^
	Dihomo-*γ*-linolenic acid (DGLA)	*all*-*cis*-8,11,14-eicosatrienoic acid	20 : 3*n* − 6 or 20: 3^Δ8,11,14^
	Arachidonic acid (AA)	*all*-*cis*-5,8,11,14-eicosatetraenoic acid	20 : 4*n* − 6 or 20: 4^Δ5,8,11,14^
	Adrenic acid (DTA)	*all*-*cis*-7,10,13,16-docosatetraenoic acid	22 : 4*n* − 6 or 22: 4^Δ7,10,13,16^
	Tetracosatetraenoic acid (TTA*_n_*_ − 6_)	*all*-*cis*-9,12,15,18-tetracosatetraenoic acid	24 : 4*n* − 6 or 24: 4^Δ9,12,15,18^
	Tetracosapentaenoic acid (TPA*_n_*_ − 6_)	*all*-*cis*-6,9,12,15,18-tetracosapentaenoic acid	24 : 5*n* − 6 or 24: 6^Δ6,9,12,15,18^
	Docosapentaenoic acid (DPA*_n_*_ − 6_)	*all*-*cis*-4,7,10,13,16-docosapentaenoic acid	22 : 5*n* − 6 or 22: 5^Δ4,7,10,13,16^

**Table 2:** The effects of various plants that contain the main omega-6 content on broiler performance.

Dose rate	Major findings	References
Broilers supplemented with 1.5% different sources of omega-3 and omega-6 (fish oil, coconut oil, canola oil, or a mixture of the three oils)	Enhanced growth performance and immune status, improved blood lipid profile and antioxidants status, and the effect of the oil sources depends on the criteria of response	Attia et al. [3]
Dietary base supplemented with 6% of the following oils: palm oil (PO), soybean oil (SO), and linseed oil (LO)	Had higher oxidative stability and cholesterol	Abdulla et al. [5]
The broilers are fed 2% of various types of omega-3 and omega-6 fatty acids (2% flax seed oil in particular)	Reduce late embryonic mortality	Saber and Kutlu [52]
Sunflower meal is added to broilers' food at a rate of 4%–12%	It did not affect carcass percentage and cut the yield of broilers	Sangsoponjit et al. [53]
Broilers supplemented with sunflower oil at a rate of 2–6%	Had greater duodenum and ilium length as well as higher fat digestibility	Khatun et al. [22]
Broilers supplemented with sunflower oil at a rate of 25–100%	LDL, HDL, and weight of the thigh, breast, heart, and pancreas and abdominal fat were not affected by the type of oil fed	Karimi et al. [54]
Broilers supplemented with sunflower oil at a rate of 2.5%–3.5%	No significant differences were obtained for different parameters of growth performance, carcass parts, and traits of groups	Gaafar et al. [55]
Basal diet supplemented with safflower oil at a rate of 5–20%	Improve weight gain, feed intake, and feed conversion ratio	Malakian et al. [56]
Canola oil was added to the diets at 0–5% concentrations	Decreases the concentrations of saturated fatty acids and blood glucose in broilers	Al-Tawash et al. [57]
Basal diet supplemented with safflower oil and inositol up to 1%	Produces relatively similar intestinal weight and length, crypt depth, and the length and width of intestinal villi	Albasheer et al. [58]
Basal diet supplemented with safflower meal at a rate of 60%	Not significantly increase the performance of chicks to increase economic efficiency	Abrham et al. [59]
Basal diet supplemented with safflower oil 5–10 g/kg	Increased the goblet cell count, mucosal thickness, intraepithelium lymphocytic lick cell infiltrations, villous height, width, and crypt depth	Amer et al. [60]
Basal diet supplemented with flaxseed oil 2.5–10 g/kg	Decreased feed intake and body weight gain but increased the feed conversion ratio and also decreased total cholesterol, triglyceride, very low-density, LDL levels in serum and increased HDL. Dietary flaxseed oil treatment significantly reduced weight gain	Al-Hilali [61]
Basal diet supplemented with flaxseed oil at rate 4% and 8%	Did not show any improvement in chicken breast meat sensory quality	Stanaćev et al. [62]
Basal diet supplemented with high-oleic peanuts at a rate of 10–12%	Increase the meat produced with unsaturated fatty acids without adversely changing the protein or amino acid content of the meat generated	Toomer et al. [63]
Basal diet supplemented with 0.1–2.0 mg/kg at the rate of *Silybum marianum*	Greater anabolic activities in their bodies and increased use of albumin fraction proteins as the principal material for organogenesis	Bagno et al. [64]
Basal diet supplemented with *Silybum marianum* at a rate of 12%	Improve broiler performance	Shahsavan et al. [65]
Basal diet supplemented with fermented rice bran and unfermented rice bran at 500 and 250 g/kg	Beneficial effect on weight gain and feed intake	Nalle and Yowi [66]
Basal diet supplemented with pumpkin seed meal at a rate of 10%	Not changing the productive performance and the sensorial quality of the meat	Martínez et al. [67]
Basal diet supplemented with extruded rice bran at a rate of 30%	Improved the broiler performance	Zare-Sheibani et al. [68]
Basal diet supplemented with *Silybum marianum* at rate 2%–3%	Achieve the maximum body weight at the lowest feed conversion per unit of body weight gain without affecting muscularity or fattening grade	Janocha et al. [69]
Basal diet supplemented with 33–100 g/kg squash seed meal	Enhanced performance and boosted edible carcass sections while decreasing belly fat in the carcass	Aguilar et al. [70]

## Data Availability

The data presented in this study are available in this article and can be accessed online or by contacting the corresponding author.

## References

[1] Brankovic Lazic I., Markovic R., Baltic B. (2021). Examination of the influence of conjugated linoleic acid in broiler nutrition on the economic efficiency of fattening. *IOP Conference Series: Earth and Environmental Science*.

[2] Sampath V., Park J. H., Kim I. H. (2020). Inclusion of dietary palm oil and soy oil on growth performance and nutrition digestibility in broiler chickens. *Korean J Poult Sci*.

[3] Attia Y. A., Al-Harthi M. A., Abo El-Maaty H. M. (2020). The effects of different oil sources on performance, digestive enzymes, carcass traits, biochemical, immunological, antioxidant, and morphometric responses of broiler chicks. *Frontiers in Veterinary Science*.

[4] Agah M. J., Nassiri-Mo H., Tahmasbi A. M., Lotfollahi H. (2010). Performance and fatty acid compositions of yolk lipid from laying hens fed with locally produced canola seed (*Brassica napus L*.). *Research Journal of Biological Sciences*.

[5] Abdulla N. R., Loh T. C., Akit H. (2015). Fatty acid profile, cholesterol and oxidative status in broiler chicken breast muscle fed different dietary oil sources and calcium levels. *South African Journal of Animal Science*.

[6] Takahashi H., Rikimaru K., Kiyohara R., Yamaguchi S. (2012). Effect of arachidonic acid-enriched oil diet supplementation on the taste of broiler meat. *Asian-Australasian Journal of Animal Sciences*.

[7] Butler G. (2014). Manipulating dietary PUFA in animal feed: implications for human health. *Proceedings of the Nutrition Society*.

[8] Watanabe N., Masubuchi D., Itoh M., Teradu S., Yazawa H., Uemura H. (2014). Oral administration of whole dihomo-*γ*-linolenic acid-producing Saccharomyces cerevisiae suppresses cutaneous inflammatory responses induced by croton oil application in mice. *Applied Microbiology and Biotechnology*.

[9] Kishawy A. T. Y., Amer S. A., Abd El-Hack M. E., Saadeldin I. M., Swelum A. A. (2019). The impact of dietary linseed oil and pomegranate peel extract on broiler growth, carcass traits, serum lipid profile, and meat fatty acid, phenol, and flavonoid contents. *Asian-Australasian Journal of Animal Sciences*.

[10] Garza A. L. I., Alba C. T., Cárdenas-Pérez R. E., Camacho A., Gutierrez-Lopez M., Castro H. (2021). Fatty acid intake during perinatal periods. *Mol Nutr*.

[11] Keegan J. D., Currie D., Knox A., Moran C. A. (2019). Redressing the balance: including DHA-rich Aurantiochytrium limacinum in broiler diets increases tissue omega-3 fatty acid content and lowers the n-6:n-3 ratio. *British Poultry Science*.

[12] Fantini J., Yahi N. (2015). Brain membranes. Brain lipids in synaptic function and neurological disease clues to innovative therap. *Strat Brain Dis*.

[13] Mariamenatu A. H., Abdu E. M. (2021). Overconsumption of omega-6 polyunsaturated fatty acids (PUFAs) versus deficiency of omega-3 pufas in modern-day diets: the disturbing factor for their “Balanced antagonistic metabolic functions” in the human body. *Journal of Lipids*.

[14] Shin D. K. (2010). *Effect of Conjugated Linoleic Acid or Oleic Acid Addition on Fatty Acid Composition Profiles of Poultry Meat*.

[15] Siram K., Rahman S. M. H., Balakumar K., Duganath N., Chandrasekar R., Hariprasad R. (2019). Pharmaceutical nanotechnology: brief perspective on lipid drug delivery and its current scenario. *Bio App Nano*.

[16] Trembeck L., Hascik P., Čuboň J., Bobko M., Pavelkova A. (2016). Fatty acids profile of breast and thigh muscles of broiler chickens fed diets with propolis and probiotics. *Journal of Central European Agriculture*.

[17] Shurson G. C., Kerr B. J., Hanson A. R. (2015). Evaluating the quality of feed fats and oils and their effects on pig growth performance. *Journal of Animal Science and Biotechnology*.

[18] Benjamin S., Prakasan P., Sreedharan S., Wright A. D. G., Spener F. (2015). Pros and cons of CLA consumption: an insight from clinical evidences. *Nutrition and Metabolism*.

[19] Milićević D., Vranić D., Mašić Z. (2014). The role of total fats, saturated/unsaturated fatty acids and cholesterol content in chicken meat as cardiovascular risk factors. *Lipids in Health and Disease*.

[20] Alagawany M., Elnesr S. S., Farag M. R. (2019). Omega-3 and Omega-6 fatty acids in poultry nutrition: effect on production performance and health. *Animals*.

[21] Cherian G. (2015). Nutrition and metabolism in poultry: role of lipids in early diet. *Journal of Animal Science and Biotechnology*.

[22] Khatun J., Loh T. C., Akit H., Foo H. L., Mohamad R. (2018). Influence of different sources of oil on performance, meat quality, gut morphology, ileal digestibility and serum lipid profile in broilers. *Journal of Applied Animal Research*.

[23] Mentang F., Jintasataporn O., Ohshima T. (2013). Fatty acid composition and sensory evaluation of the meat of broilers fed silkworm (*Bombyx mori L*) pupa dietary supplementation. *Animal Production*.

[24] Al-Khalaifah H. (2020). Modulatory effect of dietary polyunsaturated fatty acids on immunity, represented by phagocytic activity. *Frontiers in Veterinary Science*.

[25] Prasad A., Ahs M., Goncharov A., Carpenter D. O. (2010). Omega-3 and Omega-6 fatty acids kill thymocytes and increase membrane fluidity. *The Open Cell Development and Biology Journal*.

[26] Certık M., Klempova T., Guothova L., Mihalik D., Kraic J. (2013). Biotechnology for the functional improvement of cereal‐based materials enriched with PUFA and pigments. *European Journal of Lipid Science and Technology*.

[27] Laoteng K., Certik M., Cheevadhanark S. (2011). Mechanisms controlling lipid accumulation and polyunsaturated fatty acid synthesis in oleaginous fungi. *Chemical Papers*.

[28] Thanabalan A., Kiarie E. G. (2021). Influence of feeding omega-3 polyunsaturated fatty acids to broiler breeders on indices of immunocompetence, gastrointestinal, and skeletal development in broiler chickens. *Frontiers in Veterinary Science*.

[29] Ebrahim R., Liang J. B., Jahromi M. F. (2015). Effects of tannic acid on performance and fatty acid composition of breast muscle in broiler chickens under heat stress. *Italian Journal of Animal Science*.

[30] Gómez Candela C., Bermejo López L. M., Loria Kohen V. (2011). Importance of a balanced omega 6/omega 3 ratio for the maintenance of health. Nutritional recommendations. *Nutricion Hospitalaria*.

[31] Long S., Liu S., Wu D., Mahfuz S., Piao X. (2020). Effects of dietary fatty acids from different sources on growth performance, meat quality, muscle fatty acid deposition, and antioxidant capacity in broilers. *Animals*.

[32] Smink M. (2012). *Fatty Acid Digestion, Synthesis and Metabolism in Broiler Chickens and Pigs [Thesis]*.

[33] Fejerčáková A., Vašková J., Bača M. (2014). Effect of dietary microbially produced gamma-linolenic acid and plant extracts on enzymatic and non-enzymatic antioxidants in various broiler chicken organs. *Journal of Animal Physiology and Animal Nutrition*.

[34] Nilsen D. W. T., Myhre P. L., Kalstad A., Schmidt E. B., Arnesen H., Seljeflot I. (2021). Serum levels of dihomo-Gamma (*γ*)-linolenic acid (DGLA) are inversely associated with linoleic acid and total death in elderly patients with a recent myocardial infarction. *Nutrients*.

[35] Velasco S., Ortiz L. T., Alzueta C., Rebole A., Trevino J., Rodriguez M. L. (2010). Effect of inulin supplementation and dietary fat source on performance, blood serum metabolites, liver lipids, abdominal fat deposition, and tissue fatty acid composition in broiler chickens. *Poultry Science*.

[36] Gaad A. H., Shabir Barham G., Shah A. H. (2016). Effect of linoleic acid supplimentation on growth of broiler. *IOSR Journal of Agriculture and Veterinary Science*.

[37] Jahan M. S, Asaduzzaman M, Sarkar A. K (2006). Performance of broiler fed on mash, pellet and crumble. *International Journal of Poultry Science*.

[38] Pirzado S. A., Pirzado A. S., Mangsi G. S., Barham G. M., Mari Z., Pirzado (2015). Effect of mash and crumbled feed forms on the performance of broiler chickens. *J Agri Vet Sci*.

[39] Kunová S., Čuboň J., Bebejová A. (2016). Feeding effect of the addition of linoleic acid on meat quality of chickens. *Acta Universitatis Agriculturae et Silviculturae Mendelianae Brunensis*.

[40] Kanakri K., Carragher J., Hughes R., Muhlhausle B., de Koning C., Gibson R. (2017). The fatty acid composition of excreta of broiler chickens fed different dietary fatty acids. *International Journal of Poultry Science*.

[41] Pertiwi H., Mustofa I., Hernawati T. (2017). Corn oil suplementation in feed increasefolicullar hierarchy and productivity of Japanese quail. *Jurnal Veteriner*.

[42] Pertiwi H., Dadi T. (2019). Dietary of maize oil on folicullar hierarchy and visceras weight of quail (*Cortunixcortunix japonica*). *Indian Veterinary Journal*.

[43] Pertiwi H., Rochmi S. E., Dadi T. B. (2019). Blood parameter profile and helminthiasis identificationon in sheep fed with diets rich in polyunsaturated fatty acid (PUFA). *Indian Veterinary Journal*.

[44] Jung S., Choe J. H., Kim B., Yun H., Kruk Z. A., Jo C. (2010). Effect of dietary mixture of gallic acid and linoleic acid on antioxidative potential and quality of breast meat from broilers. *Meat Science*.

[45] Qi K. K., Chen J. L., Zhao G. P., Zheng M. Q., Wen J. (2010). Effect of dietary omega6/omega3 on growth performance, carcass traits, meat quality and fatty acid profiles of Beijing-you chicken. *Journal of Animal Physiology and Animal Nutrition*.

[46] Haščík P., Čuboň J., Bebejová A., Šmýkalová H., Kačániová M. (2015). Chicken carcass structure fed with addition of linoleic acid. *Journal of Microbiology, Biotechnology and Food Sciences*.

[47] Bača M., Marcinčák S., Čertík M. (2014). Effect of adding prefermented cereal product containing gamma-linolenic acid to broiler feed on production indicators and fatty acid profile of chicken breast. *Acta Veterinaria Brno*.

[48] Oliveira D. D., Baiao N. C., Cancado S. V. (2010). Effects of lipid sources in the diet of laying hens on the fatty acid profiles of egg yolks. *Poultry Science*.

[49] Khatibjoo A., Kermanshahi H., Golian A., Zaghari M. (2018). The effect of n-6/n-3 fatty acid ratios on broiler breeder performance, hatchability, fatty acid profile and reproduction. *Journal of Animal Physiology and Animal Nutrition*.

[50] El-Zenary A. S., Michael Hulet R., Ying Y., Harvatine K. J., Elkin R. G., Elkin R. G. (2020). Effect of lowering the amount of dietary linoleic acid on tissue omega-3 fatty acid contents of broilers fed supplemental flaxseed oil from 18 to 35 days of age. *The Journal of Applied Poultry Research*.

[51] Liu P., Shah B. P., Croasdell S., Gilbertson T. A. (2011). Transient receptor potential channel type M5 is essential for fat taste. *Journal of Neuroscience*.

[52] Saber S. N., Kutlu H. R. (2020). Effect of including n-3/n-6 fatty acid feed sources in diet on fertility and hatchability of broiler breeders and post-hatch performance and carcass parameters of progeny. *Asian-Australasian Journal of Animal Sciences*.

[53] Sangsoponjit S., Suphalucksana W., Srikijkasemwat K. (2017). Effect of feeding sunflower meal on the performance and carcass characteristics of broiler chickens. *Chem Engin Trans*.

[54] Karimi S. H., Zarei A., Ila N., Lotfollahian H. (2010). The effect of replacement for different levels of sunflower oil instead of soybean oil in broiler performance. *Indian Journal of Animal Research*.

[55] Gaafar H. M. A., Ayat A. R., El-Reidy K. F. A. (2014). Effect of diet supplemented with pumpkin (Cucurbita moschata) and black seed (Nigella sativa) oils on performance of rabbits: 1-Growth performance, blood haematology and carcass traits of growing rabbits. *Report Opin*.

[56] Malakian M., Hassanabad A., Heidariniy A. (2011). Effects of safflower seed on performance, carcass traits and blood parameters of broilers. *Research Journal of Poultry Sciences*.

[57] Al-Tawash A. S. A., Al-Bachry W. S. J., Al-Khaikan S. A. M. (2020). Effects of canola oil on fatty acids and biochemical traits of blood plasma in broiler chickens. *International Journal of Poultry Science*.

[58] Albasheer M. A., Iriyanti N., Ismoyowati I., Rimbawanto E. A. (2021). The balancing of safflower oil and inositol to intestinal morphometric of sentul chicken. *Animal Production*.

[59] Abrham Z., Awuk A., Teshager N., Wondifraw Z. (2018). Effects of substituting safflower (*Carthamus tinctorius*) meal with soya bean meal on the performance of SASSO X RIR crossbred chicken. *Poult Fish Wildl Sci*.

[60] Amer S. A, Mohamed W. A. M., Gharib H. S. A. (2021). Changes in the growth, ileal digestibility, intestinal histology, behavior, fatty acid composition of the breast muscles, and blood biochemical parameters of broiler chickens by dietary inclusion of safflower oil and vitamin C. *BMC Veterinary Research*.

[61] Al-Hilali A. H. (2018). Effect of dietary flaxseed oil on growth performance and serum lipid profiles in broilers. *Pakistan Journal of Nutrition*.

[62] Stanaćev V. Ž, Milošević N., Pavlovski Z. (2014). Effects of dietary soybean, flaxseed and rapeseed oil addition on broilers meat quality. *Biotehnologija u stocarstvu*.

[63] Toomer O. T., Livingston M., Wall B. (2020). Feeding high-oleic peanuts to meat-type broiler chickens enhances the fatty acid profile of the meat produced. *Poultry Science*.

[64] Bagno O., Shevchenko S., Shevchenko A. (2021). Physiological status of broiler chickens with diets supplemented with milk thistle extract. *Veterinary World*.

[65] Shahsavan M., Salari S., Ghorbani M. (2021). Effect of dietary inclusion of *Silybum marianum* oil extraction byproduct on growth performance, immune response and cecal microbial population of broiler chicken. *Biotechnology in Animal Husbandry*.

[66] Nalle C. L., Yowi M. R. K. (2019). Nutritional value of fermented rice bran for broiler chickens: apparent metabolizable energy and growth performance. *International Journal of Poultry Science*.

[67] Martínez Y., Valdivié M., Martínez O., Estarrón M., Córdova J. (2010). Utilization of pumpkin (*Cucurbita moschata*) seed in broiler chicken diets. *Cuban Journal of Agricultural Science*.

[68] Zare-Sheibani A. A., Arab M., Zamiri M. J., Rezvani M. R., Dadpasand M., Ahmadi F. (2015). Effects of extrusion of rice bran on performance and phosphorous bioavailability in broiler chickens. *Journal Of Animal Science And Technology*.

[69] Janocha A., Milczarek A., Pietrusiak D. (2021). Impact of milk thistle (*Silybum marianum L. Gaertn.*) seeds in broiler chicken diets on rearing results, carcass composition, and meat quality. *Animals*.

[70] Aguilar Y. M., Yero O. M., Navarro M. I. V., Hurtado C. A. B., L ´opez J. A. C., Mejía L. B. G. (2011). Effect of squash seed meal (*Cucurbita moschata*) on broiler performance, sensory meat quality, and blood lipid profile. *Revista Brasileira de Ciência Avícola*.

[71] Calder P. C. (2001). Polyunsaturated fatty acids, inflammation, and immunity. *Lipids*.

[72] Shearer G. C., Savinova O. V., Harries W. S. (2021). Fish oil-how does it reduce plasma trigycerides? Biochimicaet Biophysica Acta (BBA). *Mol Cell Biol Lipids*.

[73] Sidik R., Rachmawati K., Sabdoningrum E. K., Pertiwi H., Dadi T. B. (2019). The profile of cholesterol, lipoprotein, and triglyceride of blood serum of filial etawah goat fed with omega-3 rich diet. *Indian Veterinary Journal*.

[74] Bartkovský M., Mudroňová D., Marcinčáková D. (2020). Effect of fungal solid-state fermented product enriched with gamma-linolenic acid and ß-carotene on blood biochemistry and immunology of broiler chickens. *Polish Journal of Veterinary Sciences*.

[75] Jameel Y. J., Sahib A. M., Husain M. A. (2015). Effect of dietary omega-3 fatty acid on antibody production against Newcastle disease in broilers. *Int J Sci Nat*.

[76] Chhabra R., Forrester A., Lemiere S., Awad F., Chantrey J., Ganapathy K. (2015). Mucosal, cellular, and humoral immune responses induced by different live infectious bronchitis virus vaccination regimes and protection conferred against infectious bronchitis virus Q1 strain. *Clinical and Vaccine Immunology*.

[77] Kovalík P., Mačanga J., Klempová T. (2018). Effect of feeding of 5% prefermented cereal-based bioproduct enriched with *γ*-linolenic acid on production indicators, chemical composition, fatty acid profile and lipid oxidation of broiler meat. *Italian Journal of Animal Science*.

[78] Niu S., Wang C. X., Jia F. J. (2019). The expression of prostaglandinsrelated genes in erythrocytes of broiler chicken responds to thiram-induced tibial dyschondroplasia and recombinant glutathione-S-transferase A3 protein. *Research in Veterinary Science*.

[79] Calder P. C. (2010). Omega-3 fatty acids and inflammatory processes. *Nutrients*.

[80] Qadir M. F., Han X. Y., Qiao M. L. (2021). Expression of prostaglandins-related genes in erythrocytes of chickens infected with H9N2 subtype of avian influenza virus. *Pakistan Journal of Zoology*.

[81] Park S. O., Hwangbo J., Yuh I. S., Park B. S. (2014). Gamma-linolenic acid egg production enriched with hemp seed oil and evening primrose oil in diet of laying hens. *Journal of Environmental Biology*.

[82] Haug A., Olesen I., Christophersen O. A. (2010). Individual variation and intraclass correlation in arachidonic acid and eicosapentaenoic acid in chicken muscle. *Lipids in Health and Disease*.

[83] Schmitz G., Ecker J. (2008). The opposing effects of n-3 and n-6 fatty acids. *Progress in Lipid Research*.

[84] Leung H. H., Ng A. L., Durand T. (2019). Increase in omega-6 and decrease in omega-3 polyunsaturated fatty acid oxidation elevates the risk of exudative AMD development in adults with Chinese diet. *Free Radical Biology and Medicine*.

[85] Sumiati, Darmawan A., Wiryawan K. G. (2016). Egg quality and blood hematology of Magelang laying duck fed with diets containing different ratios of omega 3 and omega 6 fatty acids and organic Zn. *International Journal of Poultry Science*.

